# Periodically Pulsed Polarization Gas Sensors Based on Au|YSZ: Mechanism of NO_x_ Detection

**DOI:** 10.3390/s26072280

**Published:** 2026-04-07

**Authors:** Nils Donker, Jens Zosel, Ralf Moos, Daniela Schönauer-Kamin

**Affiliations:** 1 Department of Functional Materials, University of Bayreuth, Universitätsstraße 30, 95447 Bayreuth, Germany; 2 Kurt Schwabe Institute for Sensor Technologies (KSI), Kurt-Schwabe-Straße 4, 04736 Waldheim, Germany

**Keywords:** pulsed polarization, NO_x_-Sensor, Au|YSZ, exhaust gas sensor

## Abstract

**Highlights:**

**What are the main findings?**

- Pulsed polarization with Au electrodes on YSZ shows that NO_2_ accelerates self-discharge from the beginning, while NO, CO, and H_2_ slow down discharge in the late stage. C_3_H_6_ does not affect the discharging behavior.
- A lower O_2_ content slows down discharge and intensifies the NO_2_ effect.

**What are the implications of the main findings?**

- Oxygen supply and surface exchange at the triple-phase boundary are rate-determining during pulsed polarization.
- NO and NO_2_ might be selectively distinguished from each other by choosing appropriate electrode materials.

**Abstract:**

Pulsed polarization of Au|YSZ gas sensors is examined to clarify the mechanism of NO_x_ detection under dynamic operation and to disentangle catalytic surface effects from electrochemical relaxation. Using gold electrodes with substantially lower catalytic activity than platinum explicitly enables this mechanistic separation. During pulsed polarization, periodic voltage pulses are followed by self-discharge under open-circuit conditions, and the response is measured based on the self-discharge rate. NO_2_ consistently accelerates the self-discharge from the beginning, whereas NO slows the relaxation predominantly at later times. CO and H_2_ produce similar delaying effects, and C_3_H_6_ shows no measurable influence under the tested conditions. Decreasing ambient O_2_ slows the discharge and amplifies the NO_2_ effect, which indicates that oxygen supply and surface exchange at the triple-phase boundary are rate determining. A Pt-containing catalytic overlayer drives local NO/NO_2_ interconversion toward equilibrium so that both gases yield to an accelerated self-discharge. These findings support a mechanistic picture in which NO_2_ provides effective oxygen equivalents that accelerate discharge, whereas NO, CO, and H_2_ consume oxygen and slow down discharge. Overall, this establishes a materials-based approach for distinguishing between NO and NO_2_ and evaluating the underlying mechanism during pulsed polarization.

## 1. Introduction

Combustion processes worldwide generate substantial emissions of nitrogen oxides (NO_x_ = nitrogen monoxide (NO) and nitrogen dioxide (NO_2_)). These emissions must be monitored reliably for environmental and public health reasons [1,2,3]. Large stationary installations can rely on complex and costly analytical methods. In mobile cost-driven applications, as in the transportation sector, such solutions are often not practical.

Electrochemical high-temperature sensors offer a compact and robust alternative [4,5,6]. Established concepts include the well-known lambda probe [7,8,9,10], amperometric sensors [9,11,12,13,14,15,16,17,18,19] and sensors based on the mixed-potential principle [20,21,22,23,24,25,26,27,28,29,30,31,32,33,34,35,36]. To date, research has often focused on optimizing sensor materials to increase sensitivity and selectivity [5,29,37,38,39,40].

More recently, dynamic operating modes have come into focus, in which an input variable is deliberately modulated, and the system response is evaluated. Examples include temperature modulation [41,42,43,44,45,46,47], voltage modulation [48,49,50,51], and frequency-dependent excitation [52,53,54,55,56]. The aim of these approaches is to expand the operating window and improve selectivity with respect to interfering gases.

A particularly promising method is pulsed polarization using the Pt|YSZ system, which exhibits selective sensitivity to NO and NO_2_ [10,57,58,59,60,61]. However, the underlying mechanisms and influencing factors are not yet fully understood. This particularly concerns the discrimination between NO and NO_2_ [10]. Platinum is a catalytically active material. As a result, depending on temperature and gas composition, NO_x_ species impinging on the electrode can be converted towards a local equilibrium between NO and NO_2_ [62,63].

The objective of this work is to investigate pulsed polarization at the Au|YSZ system to gain a better understanding of the underlying mechanisms. Gold is substantially less catalytically active than platinum [62,64]. This facilitates a clearer separation of catalytic surface reactions from the electrochemical contributions to the sensor response. Unlike prior pulsed-polarization studies that predominantly used Pt|YSZ and thus convolved catalytic NO/NO_2_ equilibration with electrochemical relaxation, we deliberately employ Au|YSZ to suppress surface catalysis and explicitly decouple these contributions. Using a Pt-containing catalytic overlayer as a control further validates this mechanism.

## 2. Materials and Methods

### 2.1. Sensor Fabrication

**Figure 1:**
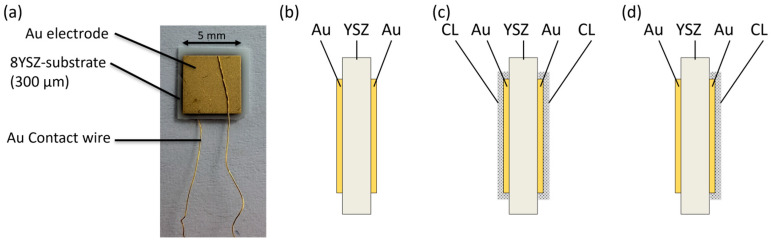
(**a**) Photograph of the fabricated Au∣YSZ∣Au sensor and (**b**) schematic cross-section showing the 8YSZ electrolyte and the screen-printed gold electrodes (**c**) with a catalytic layer (CL) on both (**d**) or on one electrode.

For sensor fabrication, a 300 µm thick ceramic with an area of approx. 6 × 6 mm^2^ made of 8 mol% yttria-stabilized zirconia (8YSZ, Kerafol GmbH, Eschenbach in der Oberpfalz, Germany) was used as the substrate, which serves as an oxide ion conductor. On both sides of the substrate, square electrode structures of 5 × 5 mm^2^ with a thickness of approximately 6 µm were applied sequentially by screen printing using a frit-free gold paste (8880-G, Ferro Corporation, Mayfield Heights, OH, USA). After each printing step, the electrodes were fired in air with a peak temperature of 850 °C. The sensors were then contacted with 100 µm gold wires by gap welding. A typical sensor is shown in Figure 1a. A schematic cross section is displayed schematically in Figure 1b.

Subsequently, some of the sensors were coated on one side (Figure 1d) or on both sides (Figure 1c) with a catalytic layer (CL), consisting of 1 wt% finely dispersed Pt in porous Al_2_O_3_, applied by brushing, in order to enable a comparison with platinum electrodes [65]. Due to the application process, the layer thickness of the CL is not constant. However, it is approximately 90 µm. These layers were annealed in air with a peak temperature of 700 °C. To exclude effects of the additional thermal step, the sensors to be compared were fired together in each case. Sensors without an additional layer underwent the same sintering cycles at 700 °C. In total, these sensors used for Section 3.5 received two additional sintering steps.

### 2.2. Pulsed Polarization

**Figure 2:**
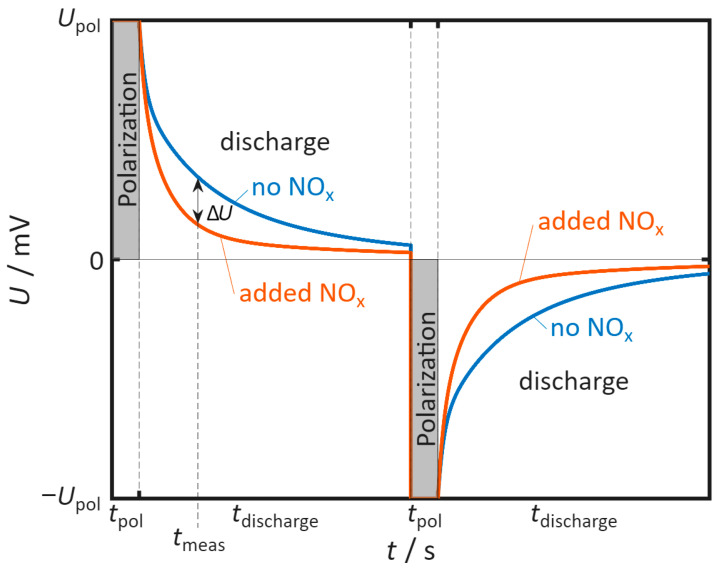
Schematic of the pulsed polarization sequence with alternating positive and negative pulses followed by open-circuit self-discharge, and reference behavior for Pt∣YSZ sensors where NO_x_ accelerates the discharge. *t*_meas_ is the point in time at which the voltage for the sensor signal is determined for each cycle. NO_x_ denotes the sum of NO and NO_2_.

In pulsed polarization, the sensor (typically Pt|YSZ|Pt and here Au|YSZ|Au) is periodically and alternately polarized with a polarization voltage *U*_pol_ for the polarization time *t*_pol_. After each pulse, the self-discharge voltage of the sensor is recorded for the discharge time *t*_discharge_. This is followed by a further polarization with reversed sign and another discharge. This sequence of polarization and discharge steps continues continuously. For the Pt|YSZ|Pt system, it has been observed that NO_x_, which is added in small concentrations to the base gas, accelerates the self-discharge of the sensor (see Figure 2). A symmetrical sensor design results in symmetrical discharge behavior in both polarization directions.

In this work, a polarization voltage *U*_pol_ of 1 V, a polarization time *t*_pol_ of 1 s, and a discharge time *t*_discharge_ of 10 s are used. A complete polarization cycle therefore comprises a positive and a negative pulse, each followed by a discharge, and has a total duration of 22 s. Determining a response time would therefore not reflect the actual response behavior of the sensor.

Pulsed polarization is implemented with custom electronics that controls the pulses and records the sensor voltages. The polarization voltage is supplied by an external voltage source and is switched to the sensor via relays. The relay circuit is shown in [66]. During the discharge phases, the sensor is disconnected from the voltage source. The sensor voltage is measured continuously (sampling rate 1 kHz) with high impedance (>10 GΩ).

To evaluate the sensor behavior, the voltage at a defined time after polarization is used, for example *U*_9500ms_, that is the voltage with *t*_meas_ = 9500 ms after the positive pulse. The larger the magnitude of this voltage, the more slowly the self-discharge of the sensor proceeds.

The difference voltage ∆*U* is also calculated to represent the difference in time between a discharge with and without NO_x_ within a cycle. It is calculated as:

∆*U*(*t*) = *U*_base gas_(t) − *U*_NOx_(t) (1)

where *U*_base gas_ denotes the voltage trace during a discharge in the base gas, and *U*_NOx_ denotes the trace with additional NO_x_ in the gas atmosphere. In the positive half-cycle, a positive ∆*U* indicates an accelerated self-discharge, whereas a negative ∆*U* indicates a slowed self-discharge. In the negative half-cycle, the sign convention is reversed.

### 2.3. Gas Measurements

The sensors are measured in a sensor test facility. The gases are dosed using mass flow controllers (Brooks Instrument, Dresden, Germany) to obtain defined gas mixtures. These mixtures flow through a gas-purged tube furnace in which the sensors are located. Previous studies have determined that 400 °C is the ideal temperature for pulsed polarization [58]. Therefore, the furnace is set to 400 °C. The control variable is the temperature, which is measured by a thermocouple located close to the sensor (Eurotherm 2416, Eurotherm Germany GmbH, Limburg an der Lahn, Germany).

The base gas is a mixture of 10 vol.% oxygen (O_2_), 2 vol.% water vapor (H_2_O), and nitrogen (N_2_). Humidity is added via an evaporator. The total flow rate is six liters per minute, referring to the humid total gas.

Gas dosing is carried out in steps, with the sensor being exposed to the base gas between each step. The specific steps and gas concentrations used are shown in the figures. NO_x_ refers to a ratio of 1:1 of NO and NO_2_. This ratio approaches thermodynamic equilibrium at 400 °C and 10% O_2_ [62,67].

## 3. Results

### 3.1. OCV Measurements

For sensors with gold electrodes, we first measure the open-circuit voltage (OCV) under NO and NO_2_. During fabrication, the electrodes are sequentially screen-printed onto 8 mol% yttria-stabilized zirconia (8YSZ) and fired. Consequently, the first-printed electrode undergoes two firing cycles, whereas the second is fired only once, which may affect its microstructure relative to the other. Because microstructure can significantly influence sensor properties, we examine the OCV between the two electrodes under varying NO, NO_2_, and mixed NO_x_ conditions [68,69].

**Figure 3:**
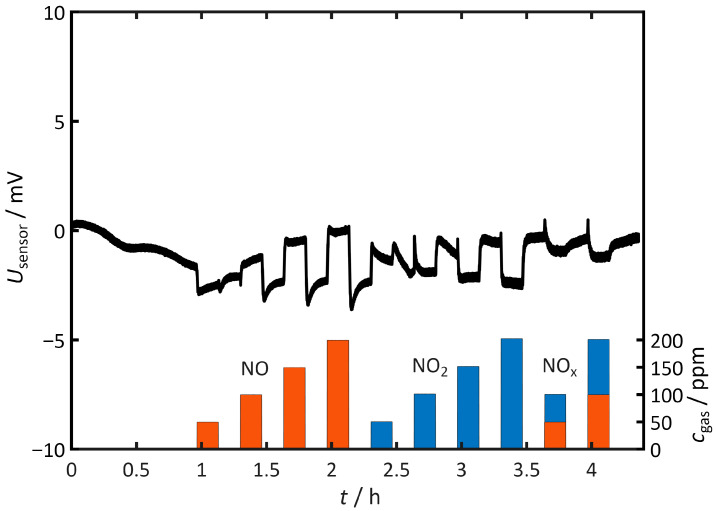
Open-circuit voltage (OCV) between the two uncovered gold electrodes under NO, NO_2_, and NO_x_. Measurement conditions: *T* = 400 °C, *c*_O2_ = 10%, *c*_H2O_ = 2%.

Figure 3 shows the voltage *U*_sensor_ measured between the electrodes as a function of time. The open-circuit voltage fluctuates within a few millivolts. The addition of NO shifts *U*_sensor_ to more positive values, while NO_2_ and NO_x_ shift *U*_sensor_ to more negative values. The absolute value remains below 5 mV. This confirms an almost symmetrical sensor in terms of reactions with nitrogen oxides. The polarization voltage *U*_pol_ of 1 V is significantly higher than the voltages measured here. Therefore, the subsequent pulsed polarization measurements can be evaluated without significant offset effects.

### 3.2. NO_x_-Dependency During Pulsed Polarization

The influence of NO_2_ and, in particular, NO on platinum electrodes during pulsed polarization has already been studied extensively. The following examines the individual effects of NO and NO_2_ on gold-electrode sensors. Additionally, the effects of an NO to NO_2_ mixture with a 1:1 ratio (NO_x_) are considered, which approximates the NO/NO_2_ ratio at thermodynamic equilibrium at 400 °C and with 10% O_2_ [62].

**Figure 4:**
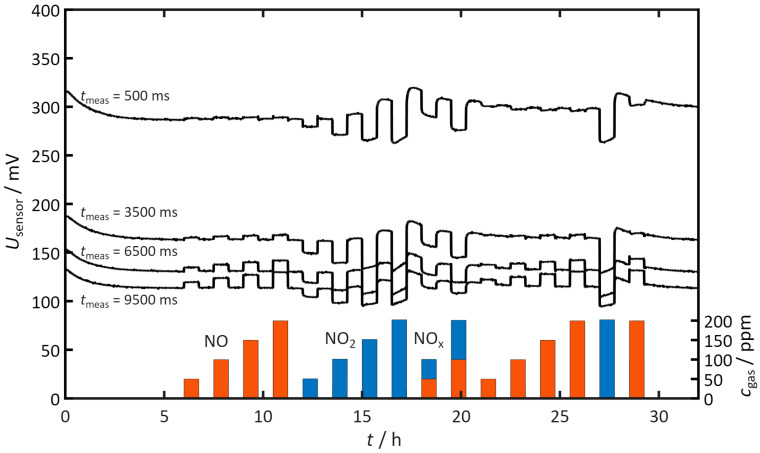
Sensor voltage *U_s_*_ensor_ at defined times after the positive pulse (*t*_meas_ = 500 ms, 3500 ms, 6500 ms, 9500 ms). The bottom right shows the dosed gas concentrations *c* for NO, NO_2_, and NO_x_ (NO/NO_2_ = 1:1). Measurement conditions: *t*_pol_ = 1 s, *t*_discharge_ = 10 s, *U*_pol_ = 1 V, *T* = 400 °C, *c*_O2_ = 10%, *c*_H2O_ = 2%.

Figure 4 shows the results of the pulsed polarization measurements. The black lines indicate the sensor voltage *U*_sensor_, which was measured 500, 3500, 6500, and 9500 ms after each positive pulse. Additionally, the dosed NO and NO_2_ concentrations are shown in the bottom.

After an initial period of about two hours, the voltages at these defined time points stabilize. This indicates that the cycles and the electrodes are highly stable after this initial phase. Therefore, after 5 h the addition of NO, NO_2_ and NO_x_ started (cf. Figure 4)

When NO is added to the gas, almost no change in *U*_sensor_ can be detected 500 ms after polarization. As the discharge progresses, the value of *U*_sensor_ increases. This is particularly observable after 6500 and 9500 ms. Self-discharge is thus slowed by NO (indicated by red bars). This effect increases with increasing NO concentration. After each NO dosing cycle is completed, the measured voltages return to their initial values. Therefore, NO affects the sensor only during exposure.

When the sensor is exposed to NO_2_ (indicated by blue bars), the measurement results show that the sensor voltages at all considered times are lower in the presence of NO_2_ than in the reference case without NO_2_. The self-discharge is therefore accelerated. The accelerating effect appears as early as about 500 ms after polarization and remains consistently present across all time points shown.

After the NO_2_ exposure ends, the voltage values rise again. Notably, however, the voltages in some cases reach higher values than before exposure to NO_2_. Furthermore, this effect becomes more pronounced with increasing NO_2_ concentration. The tendency toward slower discharge after NO_2_ exposure becomes evident during the dosing phase at a concentration of 150 ppm NO_2_ and higher.

When NO and NO_2_ are dosed simultaneously in a 1:1 ratio, more negative voltages are measured than without NO_x_. This means that faster self-discharge also occurs here. This acceleration is also more pronounced with increasing NO_x_ concentration. After the sensor has been exposed to NO_x_, it returns to its initial state.

**Figure 5:**
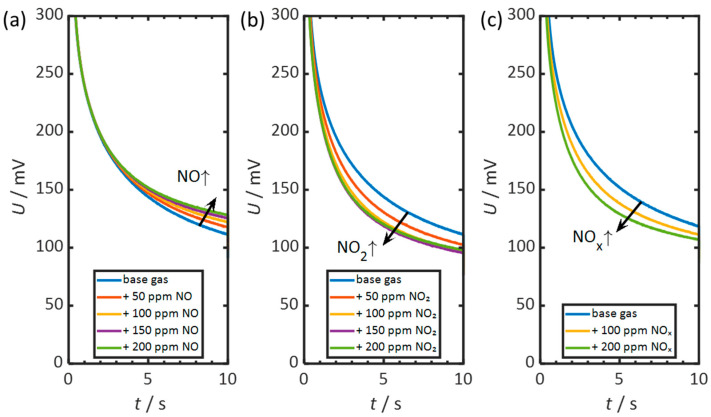
Self-discharge curves after pulsed polarization in the base gas and with the addition of (**a**) 50 to 200 ppm NO, (**b**) 50 to 200 ppm NO_2_, and (**c**) 100 and 200 ppm NO_x_ (NO/NO_2_ = 1:1). In each case the concentrations increase. Time axis: 0–10 s after the end of polarization. Measurement conditions: *t*_pol_ = 1 s, *t*_discharge_ = 10 s, *U*_pol_ = 1 V, *T* = 400 °C, *c*_O2_ = 10%, *c*_H2O_ = 2%.

For a more detailed representation, the individual discharge curves under NO, NO_2_, and NO_x_ are compared with the discharge in the base gas. The discharge curves are shown in Figure 5. Figure 5a shows the discharge with NO, Figure 5b with NO_2_, and Figure 5c with NO_x_. The curves shown originate from the measurement presented in Figure 4.

In Figure 5a, the curves with and without NO are almost identical up to about three seconds after the end of polarization. Then they diverge. With increasing NO concentration, the sensor discharges more slowly, and the voltage shifts to more positive values.

In Figure 5b, NO_2_ leads to a marked acceleration from the beginning of the discharge. The difference from the base-gas curve increases up to about three seconds and then remains approximately constant. However, starting at 100 ppm NO_2_, the discharge curves hardly differ from each other. These small differences indicate a saturation behavior in the sensor discharge. A further increasing NO_2_ concentration no longer accelerates the discharge.

Similarly, in Figure 5c, NO_x_ leads to accelerated self-discharge of the sensor. This acceleration is visible from the beginning. As the process progresses, the curve approaches that of a discharge in the base gas. This indicates the effects of NO and NO_2_ superimposing.

In summary, it can be said that NO_2_ accelerates the initial stage of self-discharge. Conversely, NO slows down self-discharge only at a later stage. An NO_x_ ratio of 1:1 causes accelerated discharge that approaches the end of the base gas curve. This behavior is similar to that of platinum electrodes, except for the effect of NO. In previous studies on Pt-based pulsed-polarization sensors, NO was generally reported to accelerate the discharge, in clear contrast to the behavior observed here for Au electrodes [10,57,58,59,60]. This acceleration reaches up to ∆*U*_0_._1s_ = 130 mV at 24 ppm NO and even ∆*U*_0_._1s_ = 250 mV at 24 ppm NO_2_ [60].

### 3.3. O_2_ Dependency

**Figure 6:**
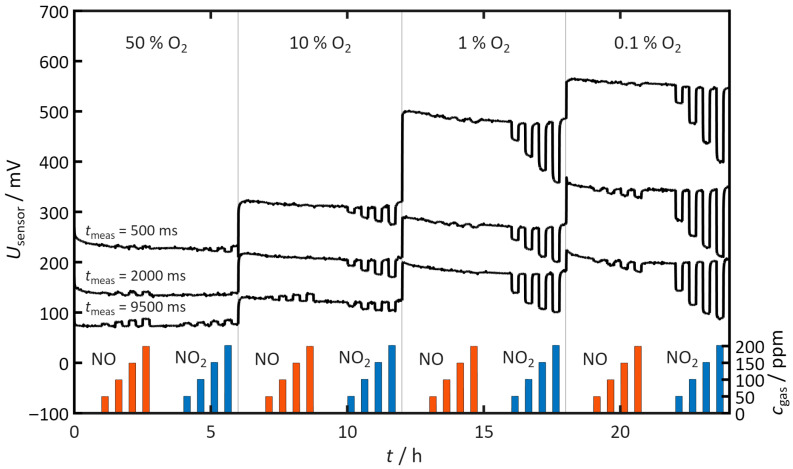
Sensor voltage *U*_sensor_ measured 500, 2000, and 9500 ms after the positive pulse at 50%, 10%, 1%, and 0.1% O_2_. Measurement conditions: *t*_pol_ = 1 s, *t*_discharge_ = 10 s, *U*_pol_ = 1 V, *T* = 400 °C, *c*_H2O_ = 2%.

Next, the influence of the oxygen content on the discharge process and on the sensor’s NO_x_ sensitivity is investigated. For this purpose, measurements are carried out at oxygen concentrations of 50%, 10%, 1%, and 0.1% O_2_. For each of these oxygen concentrations, NO and NO_2_ are dosed stepwise in the range between 50 and 200 ppm. Figure 6 shows the sensor voltage at *t*_meas_ = 500, 2000, and 9500 milliseconds after each positive polarization pulse, as well as the corresponding concentrations of the dosed NO and NO_2_ steps.

Even in operation without NO_x_ addition, it is evident that the sensor voltages increase with decreasing O_2_ content at all considered discharge times. Since the polarization voltage and pulse duration are kept constant, this increase shows that the sensor generally discharges more slowly at lower oxygen concentrations. The influence of NO and NO_2_ depends both on the respective oxygen content and on the time point within the discharge process under consideration. The following provides a detailed analysis of these effects for the individual O_2_ concentrations and discharge times.

At an oxygen content of 50%, the influence of both NO and NO_2_ is minimal. NO slightly slows the self-discharge, an effect that becomes more apparent over time. By contrast, NO_2_ causes only a slight acceleration of the discharge at the beginning, as seen at 500 ms. After 2000 ms, NO_2_ shows almost no influence. After 9500 ms, however, a slight slowing of the self-discharge occurs.

At 10% O_2_, NO shows only a slight influence on self-discharge. A slowing is visible exclusively in the late discharge region after 9500 ms. NO_2_, by contrast, accelerates the self-discharge already at the beginning of the discharge, as can be seen after 500 ms. This effect persists over the entire discharge period but decreases slightly toward the end of the discharge. The observed behavior agrees well with the results of the previous section under the same experimental conditions.

At 1% O_2_, NO leads to a very slight acceleration at the beginning of the discharge. This influence is hardly discernible after 2000 ms and no longer detectable after 9500 ms. NO_2_, in contrast, causes a continuous acceleration of self-discharge over the entire discharge time. The magnitude of this effect is higher than at 10% O_2_, thus indicating a clear increase in the effect of NO_2_ with decreasing oxygen content.

At 0.1% O_2_, NO produces an overall slightly accelerating influence on self-discharge, which is particularly pronounced at the end of the discharge period at 9500 ms. NO_2_ leads throughout the entire discharge process to a strongly pronounced accelerating effect, which is again distinctly stronger than at 1% O_2_. This shows that at this very low oxygen concentration, the strongest observed effect of NO_2_ occurs.

Overall, the discharge-accelerating influence of NO_2_ is significantly greater at all investigated oxygen concentration than that of NO. As the oxygen concentration decreases, this effect further increases, so that pronounced differences between NO and NO_2_ become visible especially at low O_2_ concentrations.

**Figure 7:**
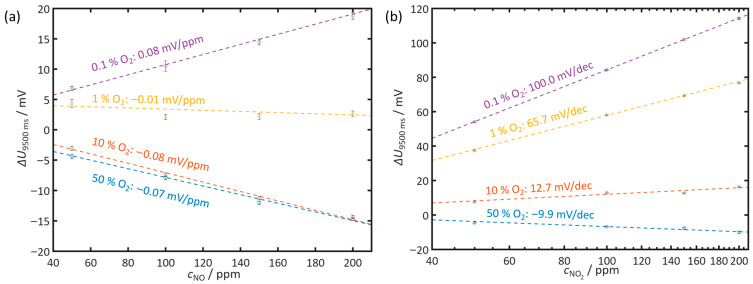
Sensor characteristic curves based on the voltage difference Δ*U*_9500ms_ for (**a**) NO and (**b**) NO_2_ at 50%, 10%, 1%, and 0.1% O_2_. Please note the linear dependence of NO and the semi-logarithmic dependence of NO_2_. The error bars indicate the standard deviation over 20 consecutive discharge cycles. Measurement conditions: *t*_pol_ = 1 s, *t*_discharge_ = 10 s, *U*_pol_ = 1 V, *T* = 400 °C, *c*_O2_ = 10%, *c*_H2O_ = 2%.

To represent the dependence of the NO and NO_2_ sensitivities on oxygen, characteristic curves are determined. For this purpose, the voltage difference Δ*U* between a discharge with and without NO_x_ is plotted against the respective NO or NO_2_ concentration (*c*_NO_, *c*_NO2_). The evaluation is carried out separately for the investigated oxygen contents of 50%, 10%, 1%, and 0.1% O_2_. For all measurements, the *U*_9500ms_ voltage, which occurs at *t*_meas_ = 9500 ms after the positive pulse, is used since changes in voltage can be reliably captured at this time for all gases and oxygen contents. The resulting characteristic curves are shown in Figure 7.

NO (Figure 7a): For NO, the sensor shows a linear dependence between Δ*U* and the NO concentration. The determined sensitivities range from −0.07 mV per ppm NO at 50% O_2_ to 0.08 mV per ppm NO at 0.1% O_2_. At 1% O_2_, the sensitivity is almost zero.

NO_2_ (Figure 7b): The characteristic curves for NO_2_ exhibit a semi-logarithmic form. Δ*U* shows a linear dependence on the logarithm of the NO_2_ concentration. The sensitivities range between −9.9 millivolts per decade NO_2_ (mV/dec) at 50% O_2_ and +100 mV/dec at 0.1% O_2_. For 1% O_2_, a sensitivity of 65.7 mV/dec is obtained. For 10% O_2_, it is 12.7 mV/dec. Interpolating the sensitivities as a function of the oxygen concentration reveals a change in sign at approximately 25% O_2_. The voltage differences Δ*U* for NO_2_ reach significantly higher values, particularly at low oxygen contents, than in the case of NO.

The measurements show that both the NO and NO_2_ characteristic curves depend strongly on the oxygen content. For NO, the sensitivities shift from slightly negative to slightly positive between 50% and 0.1% O_2_. For NO_2_, the sensitivity increases sharply with decreasing O_2_ content and thereby changes from the negative to the positive range. The four oxygen levels each produce clearly distinguishable characteristic curves with different slopes and absolute values of the voltage differences.

### 3.4. Response to Other Gases

**Figure 8:**
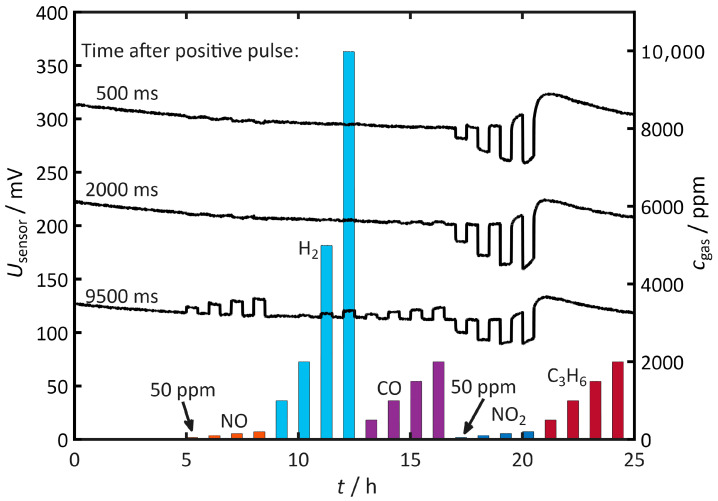
Time course of the sensor voltage *U*_sensor_ at 500 ms, 2000 ms, and 9500 ms after the positive pulse during the stepwise dosing of NO, H_2_, CO, NO_2_, and C_3_H_6_. Bars indicate the dosing steps. The right axis shows the respectively dosed concentrations. Measurement conditions: *t*_pol_ = 1 s, *t*_discharge_ = 10 s, *U*_pol_ = 1 V, *T* = 400 °C, *c*_O2_ = 10%, *c*_H2O_ = 2%.

As a further investigation, the principle of pulsed polarization is extended to additional gases. In addition to nitric oxide (NO) and nitrogen dioxide (NO_2_), hydrogen (H_2_), carbon monoxide (CO), and propene (C_3_H_6_) are included. Figure 8 shows the sensor voltage *U*_sensor_ at *t*_meas_ = 500 ms, 2000 ms, and 9500 ms after the positive pulse, as well as the stepwise dosed concentrations of the individual gases on the right axis.

With NO, the sensor has little influence on self-discharge at the beginning of the process. Only after a long discharge time of 9500 ms does the self-discharge slow down, resulting in increased voltages. This behavior corresponds to the previously described observations.

A similar trend is observed with H_2_. Its influence remains marginal at the beginning of the discharge, and the self-discharge slows again at 9500 ms. However, the effect is less pronounced than with NO, despite the significantly higher concentrations used-up to 10,000 ppm H_2_ compared to 200 ppm NO.

There is no influence on the sensor voltage observed for CO at the beginning of the discharge either. As the discharge progresses, self-discharge slows down. Its intensity, relative to the applied concentration, lies between the effects of NO and H_2_.

In contrast, NO_2_ accelerates discharge throughout the entire period. Additionally, once the concentration of NO_2_ reaches approximately 150 ppm, the voltage curves do not fully return to the level observed prior to exposure. Following NO_2_ dosing, the discharge initially proceeds at a lower rate than before exposure. At 200 ppm of NO_2_, this effect occurs during the dosing phase. Over the course of several hours, the self-discharge gradually approaches its initial value again.

No discernible influence on self-discharge is observed for C_3_H_6_. Minor changes are superimposed by the aftereffect of the previous NO_2_ dosing.

**Figure 9:**
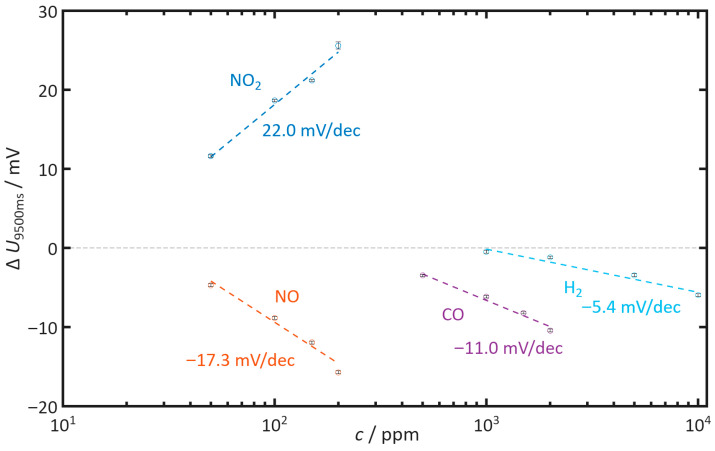
Characteristic curves of Δ*U*_9500ms_ as a function of concentration for NO, NO_2_, CO, and H_2_, evaluated 9500 ms after the end of the polarization. The error bars indicate the standard deviation over 20 consecutive discharge cycles. Measurement conditions: *t*_pol_ = 1 s, *t*_discharge_ = 10 s, *U*_pol_ = 1 V, *T* = 400 °C, *c*_O2_ = 10%, *c*_H2O_ = 2%.

Figure 9 summarizes the characteristic curves of the investigated gases. Since some gases only affect the discharge process at a late stage, the voltage differences at the end of self-discharge Δ*U*_9500ms_ were considered. Positive values correspond to accelerated self-discharge, while negative values correspond to slowed self-discharge.

Under the chosen conditions, only NO_2_ leads to an accelerated self-discharge. The determined sensitivity is 22.0 mV per decade (mV/dec) of NO_2_. NO leads to slowed self-discharge with a sensitivity of −17.3 mV/dec NO. For CO, the sensitivity is −11.0 mV/dec CO, and for H_2_, it is −5.4 mV/dec H_2_.

### 3.5. Catalytical Active Layer

Previous results show that with gold electrodes only NO_2_ accelerates the self-discharge. To reproduce the influence of platinum electrodes and examine the underlying mechanism more closely, additional sensors with gold electrodes and a catalytic layer (CL) were measured. Three variants are used: sensors without a catalytic layer, sensors with a single-sided catalytic layer, and sensors with a double-sided catalytic layer on the electrode surfaces (see Figure 1b–d). The sensors without a catalytic layer (CL) differ from the previously used sensors in that their electrodes underwent two additional firing steps to ensure better comparability with the coated variants.

**Figure 10:**
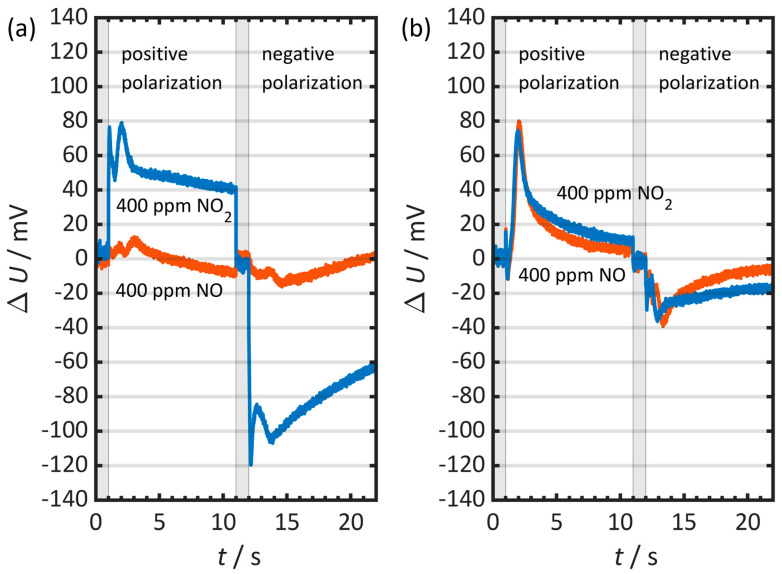
Time course of the voltage difference Δ*U* = *U*_base gas_ − *U*_NOx_ at 400 ppm NO and 400 ppm NO_2_ for (**a**) a sensor without a catalytic layer and (**b**) a sensor with a catalytically active layer on both gold electrodes. In the positive half-cycle, a positive ∆*U* indicates an accelerated self-discharge, whereas a negative ∆*U* indicates a slowed self-discharge. In the negative half-cycle, the sign convention is reversed. The gray bars represent the polarization periods. Measurement conditions: *t*_pol_ = 1 s, *t*_discharge_ = 10 s, *U*_pol_ = 1 V, *T* = 400 °C, *c*_O2_ = 10%, *c*_H2O_ = 2%.

To analyze the sensor behavior, the time course of the voltage difference Δ*U* is used. In the positive polarization direction, Δ*U* > 0 indicates faster self-discharge, while Δ*U* < 0 indicates slower self-discharge. In the negative polarization direction, this assignment is reversed in each case. Figure 10 shows the corresponding Δ*U* profiles for (a) a sensor without catalytic coating and (b) a sensor with a catalytically active layer on both gold electrodes.

Figure 10a shows that 400 ppm NO_2_ leads to a pronounced acceleration of self-discharge in both polarization directions for the uncoated sensor, whereas 400 ppm NO only slightly affects the behavior. In contrast, for the sensor coated on both sides in Figure 10b, both 400 ppm NO and 400 ppm NO_2_ cause accelerated self-discharge of the sensor. The respective Δ*U* profiles of NO and NO_2_ differ only slightly from each other.

The results show that the catalytic layer probably shifts the supplied gas mixture on its way to the electrode toward thermodynamic equilibrium. As a result, an approximately constant fraction of NO and NO_2_ is established regardless of the dosed gas composition, so that NO_2_ is present at the electrode in all cases and thus accelerated discharge occurs. Under the measurement conditions used here, the catalytic layer exhibits this behavior reproducibly.

**Figure 11:**
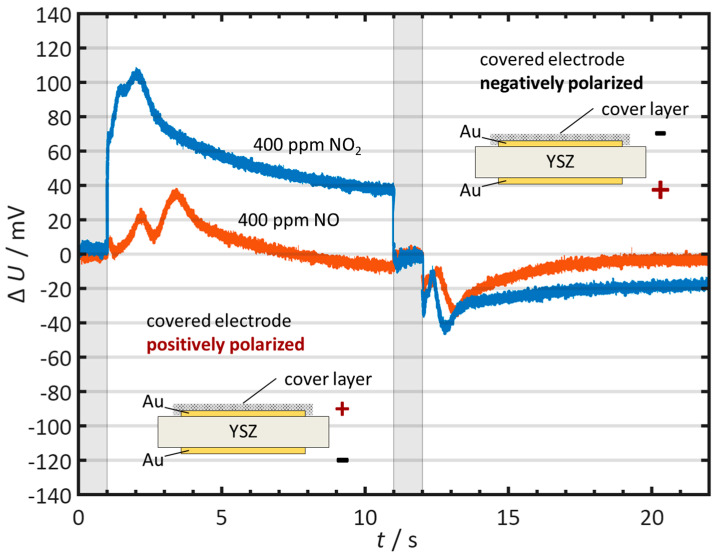
Time course of the difference voltage Δ*U* for 400 ppm NO and 400 ppm NO_2_ measured on the sensor with a single-sided catalytic coating. On the left, the uncoated electrode is negatively polarized. On the right, the coated electrode is negatively polarized. In the positive half-cycle, a positive ∆*U* indicates an accelerated self-discharge, whereas a negative ∆*U* indicates a slowed self-discharge. In the negative half-cycle, the sign convention is reversed. The gray bars represent the polarization periods. Measurement conditions: *t*_pol_ = 1 s, *t*_discharge_ = 10 s, *U*_pol_ = 1 V, *T* = 400 °C, *c*_O2_ = 10%, *c*_H2O_ = 2%.

Additionally, a sensor with a single-sided applied catalytic layer is measured (see Figure 11) to clarify the role of the two electrodes during pulse polarization. The difference voltages Δ*U*, referenced to the discharge in the base gas without NO_x_, show clear differences depending on the polarization direction.

When the uncoated electrode is negatively polarized, NO and NO_2_ can be clearly distinguished from each other. In this polarization direction, the resulting sensor behavior corresponds to that of the uncoated sensor from Figure 10a. By contrast, when the coated electrode is negatively polarized, the values of Δ*U* for NO and NO_2_ are very close to each other. This behavior corresponds to the sensor with electrodes coated on both sides in Figure 10b.

Accordingly, the sensor in each case exhibits the behavior of the negatively polarized electrode. If this electrode is coated, the sensor behavior corresponds to that of a double-sided coating; if it is uncoated, the behavior is analogous to that of the uncoated sensor. It follows that the negatively polarized electrode determines the sensor behavior in this setup.

In summary, sensors with gold electrodes and a catalytic layer applied on both sides behave like sensors with platinum electrodes. With uncoated gold electrodes, NO_2_ leads to a pronounced acceleration of the self-discharge, while NO leads only to minor effects. If only one electrode side is coated, the two polarization directions differ markedly. In this case, the negatively polarized electrode determines the sensor response to NO_x_.

## 4. Discussion

Yttria-stabilized zirconia is an oxide-ion conductor used in oxygen sensors, oxygen pumps, and fuel cells. When a voltage is applied to the sensor during the polarization period, oxide ions are transported through the solid electrolyte according to reactions (2) and (3) [70,71,72]:

O_2_ + 4e^−^ → 2O^2−^ (2)

2O^2−^ → O_2_ + 4e^−^ (3)

Here, O_2_ is an oxygen molecule, e^−^ is an electron, and O^2−^ is a doubly negatively charged oxide ion. The applied voltage causes oxygen ions to migrate through the solid electrolyte at the atomic level. As a result, different oxygen partial pressures are established during polarization at the electrodes, i.e., at the three-phase boundary. Immediately after polarization, a pronounced, artificially generated oxygen gradient is therefore present.

After the polarization is switched off, self-discharge begins. In the course of this process, the sensor returns to its thermodynamic initial state. The sensor voltage is coupled to the oxygen gradient via the Nernst equation [73]:

(4) $$U\;=\;\frac{RT}{zF}\ln\left(\frac{p_{O_{2}}^{I}}{p_{O_{2}}^{II}}\right)$$

*U* is the voltage between the electrodes. *R* is the universal gas constant. *T* is the absolute temperature in Kelvin and *z* is the number of transferred electrons. In this case *z* = 4 as shown in reactions (2) and (3). *F* is the Faraday constant. Fast effects such as ohmic losses during polarization are not considered here. These effects decay quickly and therefore influence the discharge only briefly at the beginning.

As observed, the sensor’s discharge process does not proceed abruptly. Instead, the voltage decreases gradually over several seconds. Even after ten seconds, the voltage is still above 100 mV (10% of *U*_Pol_), as shown in Figure 5. This indicates that the oxygen gradient previously generated by polarization, as well as other polarization effects such as oxidized species, have not yet been completely degraded at this point in time.

The measurements show a clear relationship between the ambient oxygen content and the rate of self-discharge. At high oxygen concentrations, relaxation proceeds faster than at low concentrations, as illustrated in Figure 6. This observation suggests that the resupply of oxygen to the oxygen-poor electrode is the rate-determining step. This step comprises gas transport to the surface, adsorption at the electrode, and electrochemical surface exchange at the three-phase boundary. If the oxygen partial pressure in the environment is high, sufficient molecular oxygen is available. It can rapidly diffuse to the electrode and adsorb there. At low oxygen partial pressure, this process is slower. The decay of the gradient slows and the sensor voltage remains elevated for longer. This finding is supported by the fact that the negative low-oxygen electrode dominates the sensor behavior with or without a catalytic layer.

Gases can influence self-discharge by either promoting the oxygen supply or competing for available oxygen species. NO_2_ accelerates the discharge over the entire relaxation period. As a strong oxidizing agent, NO_2_ effectively provides oxygen equivalents at the three-phase boundary and thus increases the effective oxygen flux for the decay of the gradient. This effect depends on the ambient oxygen partial pressure. At a high oxygen fraction, there is already sufficient oxygen present. In this case, NO_2_ provides only a limited additional contribution. At low oxygen partial pressure, the contribution of NO_2_ is significantly higher. In this case, self-discharge is more strongly accelerated. The decisive factor is likely not the total amount of oxygen, but rather the rate at which oxygen is supplied to the three-phase boundary. This rate encompasses both reaction and adsorption kinetics.

NO exhibits the opposite behavior. It hinders the discharge, especially later on when the oxygen gradient has decreased and molecular oxygen is more readily available. In this regime, nitric oxide presumably uses the available oxygen for being oxidized to NO_2_. As a result, oxygen species are bound and withdrawn from the discharge process. The resulting competition for oxygen delays the decay of the gradient. Direct decomposition of nitrogen monoxide is likely suppressed by the gold electrodes used here. This effect is also known for electrodes in amperometric oxygen sensors, where Au suppresses reactions of the nitrogen oxide system and thereby minimizes cross-sensitivities [37,74,75].

CO and H_2_ can analogously react with available oxygen species. Both gases consume oxygen through oxidation to carbon dioxide (CO_2_) or water (H_2_O) and thus compete directly with the relaxation. The observed differences in sensitivities reflect their respective reactivity and their tendency to bind oxygen and withdraw it from the discharge process.

The role of the electrode material is consistent with these results. If the observations are transferred to sensors with platinum electrodes, a consistent picture emerges. In these sensors, NO_2_ accelerates the self-discharge process more strongly than nitrogen monoxide, as has been shown for planar sensors. Only in lambda probes is the sensitivity to NO_2_ comparable to the sensitivity to nitrogen monoxide. This behavior can be explained by the catalytically active coatings of the lambda probe. These coatings accelerate the conversion between nitrogen monoxide and NO_2_ and establish thermodynamic equilibrium sufficiently quickly, so that no difference can be detected.

The temperature dependence of sensitivity to nitrogen monoxide in platinum electrodes supports this interpretation. The literature describes that sensitivity to nitrogen monoxide increases with sensor temperature up to about 400 °C and then decreases again [58]. In relation to the mechanism presented here, this means the following: At low temperatures, the surface kinetics and reaction kinetics for the formation of NO_2_ from nitrogen monoxide are too slow. Therefore, only a small amount of NO_2_ forms locally, which therefore only slightly accelerates the self-discharge. At around 400 °C, the local nitrogen dioxide concentration reaches a maximum, as the kinetics are sufficiently fast and the equilibrium between nitrogen monoxide and NO_2_ is not yet strongly biased towards nitrogen monoxide. Accordingly, the apparent sensitivity to nitrogen monoxide is at its maximum. At higher temperatures, the thermodynamic equilibrium between nitrogen monoxide and NO_2_ shifts increasingly in favor of nitrogen monoxide. As a result, the available NO_2_ concentration decreases again, which also reduces the sensitivity to nitrogen monoxide.

The slowed discharge observed after the exposure of high NO_2_ concentrations may be due to the formation of strongly bound nitrates during the polarization [76]. These bound nitrates could block adsorption sites, which are then unavailable for self-discharge. Due to the long recovery times of several hours, it is not feasible to use the sensor under these conditions at concentrations exceeding 150 ppm of NO_2_.

In summary, it can be summarized that NO_2_ probably provides effective oxygen equivalents due to its oxidizing effect and thus supports the reduction in the oxygen gradient. Nitric oxide, on the other hand, acts predominantly indirectly, as it must first be oxidized to nitrogen dioxide. The resulting NO_2_ can then be reduced again, thereby accelerating the discharge, provided that the oxygen required for this does not come from the immediate vicinity of the three-phase boundary. Acceleration through the direct decomposition of nitric oxide would only be expected at higher oxygen gradients and temperatures. In this case, the sensor temperature is low compared to amperometric NO-sensors [77,78,79]. The slowdown in self-discharge observed with nitrogen monoxide shows that, under the conditions relevant here, the reaction predominantly proceeds in the direction of oxygen consumption. Although the electrode material can significantly influence the reaction rates, it does not change the direction of the reaction. Therefore, NO_2_ is probably also the main cause of accelerated discharge in platinum electrodes.

## 5. Conclusions

Pulsed polarization in Au|YSZ sensors allows for the mechanistic separation of the effects of NO and NO_2_ on self-discharge. NO_2_ accelerates the self-discharge throughout the discharge period, while NO, CO, and H_2_ primarily decelerate the process in the late stage. Additionally, the self-discharge rate increases with higher oxygen content. These findings suggest that at the negatively polarized, low-oxygen electrode, the rate-determining steps occur. Studies with single-sided and double-sided catalytic layers demonstrate that the negatively polarized electrode defines the sensor response and that the catalytic layer modifies the behavior to resemble that of platinum electrodes. These studies have provided deeper insight into the functioning of pulsed polarization.

Although this work focuses exclusively on elucidating the underlying mechanism, this electrode configuration could also be useful in practical applications. In combustion processes, NO and NO_2_ usually occur together, which makes selective detection difficult. Combining the gold electrodes presented here with platinum electrodes could potentially combine the performance of the platinum electrodes with the NO_2_ selectivity of the gold electrodes, thus combining the advantages of both.

## Data Availability

All relevant data presented in the article are stored according to institutional requirements and as such are not available online. However, all data used in this paper can be made available upon request to the authors.

## Abbreviations

The following abbreviations are used in this manuscript:

8YSZ	8 mol-% yttria-stabilized zirconia
CL	Catalytic layer
OCV	Open-Circuit Voltage
ppm	parts per million
